# Supplementary material for: Novel Driver Strength Index highlights important cancer genes in TCGA PanCanAtlas patients
Source: PeerJ. 2022 Aug 11;10:e13860. doi: 10.7717/peerj.13860 (PMC9375969; doi:10.7717/peerj.13860)
Supplement: Supplemental Information 2 [file peerj-10-13860-s002.zip › COHORTS/genes plots/cohorts/2021_8_16_14_9_distribution_cohorts_males_THYM.pdf]

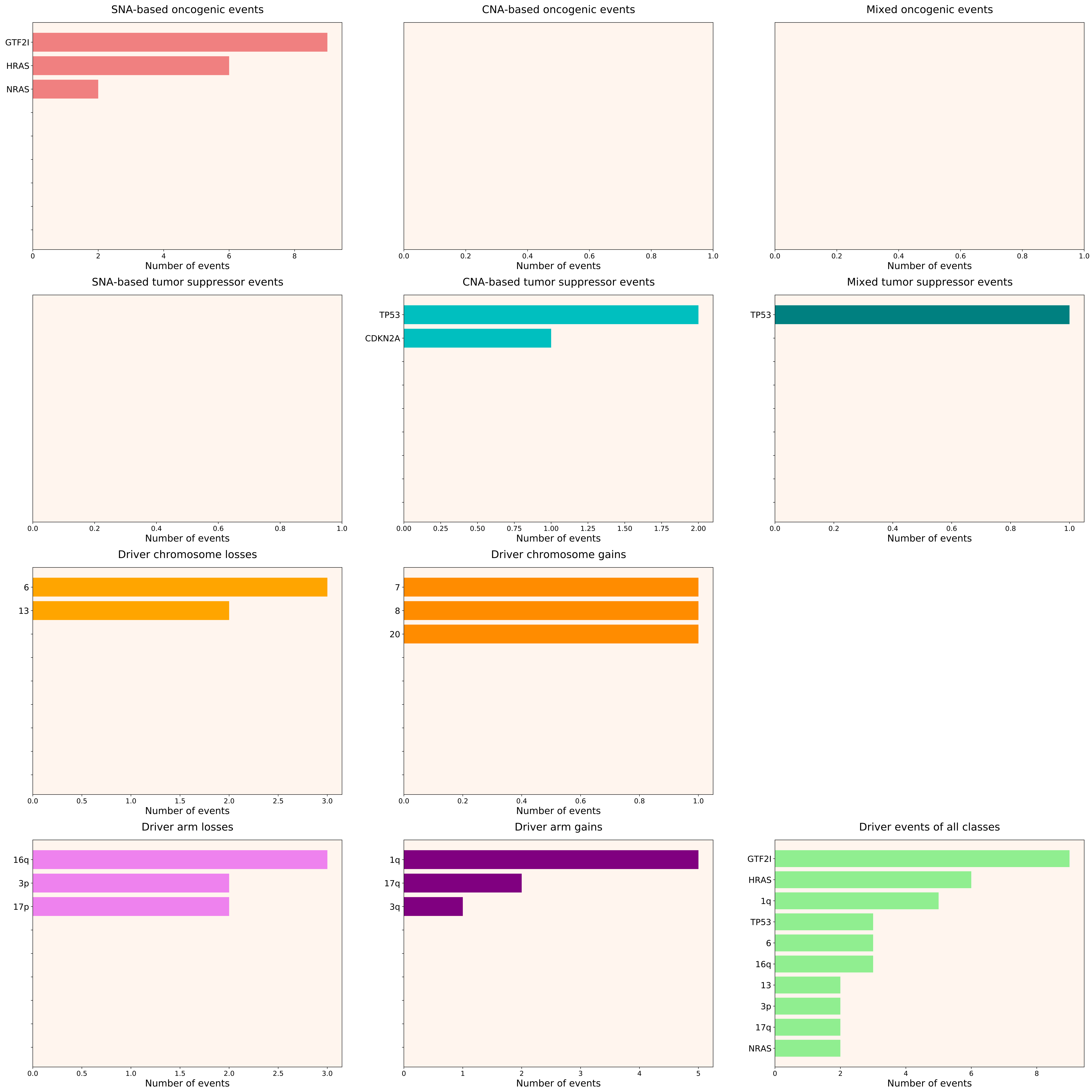

SNA-based oncogenic events
GTF2I
HRAS
NRAS
0 2 4 6 8
Number of events
CNA-based oncogenic events
0.0 0.2 0.4 0.6 0.8 1.0
Number of events
Mixed oncogenic events
0.0 0.2 0.4 0.6 0.8 1.0
Number of events
SNA-based tumor suppressor events
0.0 0.2 0.4 0.6 0.8 1.0
Number of events
CNA-based tumor suppressor events
TP53
CDKN2A
0.00 0.25 0.50 0.75 1.00 1.25 1.50 1.75 2.00
Number of events
Mixed tumor suppressor events
TP53
0.0 0.2 0.4 0.6 0.8 1.0
Number of events
Driver chromosome losses
6
13
0.0 0.5 1.0 1.5 2.0 2.5 3.0
Number of events
Driver chromosome gains
7
8
20
0.0 0.2 0.4 0.6 0.8 1.0
Number of events
Driver arm losses
16q
3p
17p
0.0 0.5 1.0 1.5 2.0 2.5 3.0
Number of events
Driver arm gains
1q
17q
3q
0 1 2 3 4 5
Number of events
Driver events of all classes
GTF2I
HRAS
1q
TP53
6
16q
13
3p
17q
NRAS
0 2 4 6 8
Number of events
